# Similar Processes but Different Environmental Filters for Soil Bacterial and Fungal Community Composition Turnover on a Broad Spatial Scale

**DOI:** 10.1371/journal.pone.0111667

**Published:** 2014-11-03

**Authors:** Nicolas Chemidlin Prévost-Bouré, Samuel Dequiedt, Jean Thioulouse, Mélanie Lelièvre, Nicolas P. A. Saby, Claudy Jolivet, Dominique Arrouays, Pierre Plassart, Philippe Lemanceau, Lionel Ranjard

**Affiliations:** 1 Unité Mixte de Recherche 1347 Agroécologie, Institut National de la Recherche Agronomique-AgroSup Dijon-Université de Bourgogne, Dijon, France; 2 Unité Mixte de Recherche 1347 Agroécologie-Plateforme GenoSol, Institut National de la Recherche Agronomique-AgroSup Dijon-Université de Bourgogne, Dijon, France; 3 Unité Mixte de Recherche 555 Laboratoire de Biométrie et Biologie Evolutive, Université Lyon 1-Centre National de la Recherche Scientifique, Villeurbanne, France; 4 Unité de Services 1106 InfoSol, Institut National de la Recherche Agronomique, Orléans, France; University of Tartu, Estonia

## Abstract

Spatial scaling of microorganisms has been demonstrated over the last decade. However, the processes and environmental filters shaping soil microbial community structure on a broad spatial scale still need to be refined and ranked. Here, we compared bacterial and fungal community composition turnovers through a biogeographical approach on the same soil sampling design at a broad spatial scale (area range: 13300 to 31000 km^2^): i) to examine their spatial structuring; ii) to investigate the relative importance of environmental selection and spatial autocorrelation in determining their community composition turnover; and iii) to identify and rank the relevant environmental filters and scales involved in their spatial variations. Molecular fingerprinting of soil bacterial and fungal communities was performed on 413 soils from four French regions of contrasting environmental heterogeneity (Landes<Burgundy≤Brittany<<South-East) using the systematic grid of French Soil Quality Monitoring Network to evaluate the communities’ composition turnovers. The relative importance of processes and filters was assessed by distance-based redundancy analysis. This study demonstrates significant community composition turnover rates for soil bacteria and fungi, which were dependent on the region. Bacterial and fungal community composition turnovers were mainly driven by environmental selection explaining from 10% to 20% of community composition variations, but spatial variables also explained 3% to 9% of total variance. These variables highlighted significant spatial autocorrelation of both communities unexplained by the environmental variables measured and could partly be explained by dispersal limitations. Although the identified filters and their hierarchy were dependent on the region and organism, selection was systematically based on a common group of environmental variables: pH, trophic resources, texture and land use. Spatial autocorrelation was also important at coarse (80 to 120 km radius) and/or medium (40 to 65 km radius) spatial scales, suggesting dispersal limitations at these scales.

## Introduction

For over two centuries, biogeographical studies have been carried out on macroorganisms and have provided a better understanding of species distribution, extinction and interactions [1]–[2]. For microorganisms, the first biogeographic postulate was developed by Baas Becking in 1934 [3]: “Everything is everywhere, *but*, the environment selects” suggesting that microbial “species” may be everywhere due to huge dispersal potentials, but that their abundances are constrained by contemporary environmental context, which may be especially true at broad spatial scales (spatial scales larger than 100 km^2^ are considered as broad in this study). The number of studies in microbial biogeography has increased exponentially over the past decade thanks to new molecular tools applicable in routine on wide scale sampling networks constituted of several hundreds of samples [4]–[5]. These studies revealed that soil microorganisms are not strictly cosmopolitan since their distributions are systematically heterogeneous and structured into biogeographical patterns [2], [6]–[9].

One way to discriminate the spatial processing of microbial diversity is to evaluate either the Taxa-Area Relationship (TAR), *i.e.* the accumulation of new taxa with increasing sampling area, or the Distance-Decay Relationship (DDR), *i.e.* the rate of change in compositional similarity with increasing distance. [10]–[11]. Although significant TAR and DDR have recently been demonstrated for both soil fungal [12] and bacterial [8], [11], [13]–[14] communities, the relative importance of the ecological processes shaping these communities is still under debate. Therefore, it needs to be more deeply considered at the community level. According to Vellend [15], four processes are involved in shaping microbial community composition: selection, dispersal, ecological drift and speciation. Speciation is difficult to consider at the community level because the molecular markers used to discriminate microbial taxa mainly target highly conserved regions (*e.g.* ribosomal genes) with low mutation rates. The stochastic demographic processes underlying ecological drift are also difficult to consider since it remains a challenge to fully characterize demographic evolutions within complex microbial communities in environmental samples. Consequently, most biogeographical studies have focused on environmental selection and dispersal limitations, the later leading to a spatial autocorrelation between sites independently of environmental factors. Numerous studies have identified environmental selection as relevant in shaping soil bacterial community composition [8]–[9], [16]–[24]. Conversely, dispersal limitation is still under debate regarding the high dispersal potentials of microorganisms and because some environmental variables always remain unmeasured. Nevertheless, recent publications also suggest that bacteria may be dispersal limited [9], [21]–[23] or that part of soil bacterial communities is endemic [17]. As regards soil fungi, the relevance of environmental selection and dispersal limitations has been demonstrated at the community level and for ectomycorrhizal groups [16], [19], [22], [24]–[29]. Nevertheless, most of these studies were performed on different sampling designs with different molecular techniques. Only few studies have investigated such processes for both soil fungal and bacterial communities simultaneously to compare their biodiversity turnover. Most of them were performed in particular ecosystems and lead to diverging conclusions: Pasternak et al [18] concluded that bacterial and fungal communities were primarily shaped by environmental selection rather than dispersal limitations at the scale of the Israeli desert. On the contrary, Talbot et al. [27] highlighted a strong endemism for fungi in pine forests and Hovatter [22] suggested that the ecological processes shaping soil bacterial community could differ at a local scale due to the presence/absence of a particular plant. Altogether, this suggests that environmental heterogeneity may determine the relative importance of the ecological processes at work and therefore affect the distance-decay relationship for both soil bacteria and fungi [9]–[11], also suggested by other macrobial studies [30]–[31]. The comparison of different microbial communities along different levels of environmental heterogeneity may therefore help to reach a consensus.

Both selection and dispersal are based on the various ecological attributes of soil bacteria and fungi in terms of soil colonization, dispersal forms, trophic requirements, biological interactions and adaptation to environmental conditions, together with stochastic factors. Consequently, studies focusing particularly on soil bacteria or soil fungi have identified numerous environmental filters involved in shaping these particular communities but no consensus could be reached regarding microbial community as a whole on broad spatial scales. The filter most frequently identified for bacteria is soil pH [5], [17], [20], [23]–[24], [32]–[34] and it is commonly assumed that this is an important driver for fungal communities [18], [24]. Soil texture and carbon content have also been identified as important filters for bacteria [6], [22]–[23], [33]–[34]. Similarly, the quality of soil organic matter, represented by the C:N ratio, and the amount of N were shown to have a significant effect on the abundance and composition of bacterial and fungal communities [24], [34]–[35]. These edaphic factors are often considered as the main determinants of bacterial diversity since the importance of climate may vary across biomes at a continental scale [5], [12], [16]. Land-use, agricultural practices, and plant community composition are also important filters for both bacteria and fungi on a wide scale [18], [19], [22]–[24], [36]–[38]. Therefore, this suggests that common filters determine the composition of both bacterial and fungal communities, but they still need to be ranked according to their relative importance to reach a consensus. This may be achieved by comparing bacterial and fungal communities over regions contrasted in terms of habitat heterogeneity but also with wide ranges of variations for the identified filters.

The objectives of this study were: i) to examine the spatial structuring of bacterial and fungal communities on a broad spatial scale; ii) to investigate the relative importance of environmental selection and spatial autocorrelation in determining the community composition turnover of these communities; and iii) to identify and rank the relevant environmental filters and scales involved in their spatial variations. To attain these objectives, four regions in the RMQS data set (“Réseau de Mesures de la Qualité des Sols” = French Monitoring Network for Soil Quality, recovering 2,200 soils over the whole of France) were selected along a gradient of environmental heterogeneity, representing a total of 413 soils. This gradient was chosen in order to confront the community composition turnover rates of bacterial and fungal communities to soil habitat heterogeneity [9]. Bacterial and fungal communities were characterized by Automated RISA fingerprinting of soil DNA. Community composition turnover (z) was estimated by means of a similarity DDR using an exponential model as suggested by Harte et al [39]–[40] for microorganisms. Together with this measure of community composition turnover over broad spatial scales, the initial similarity of communities was evaluated [30]. It represents the variability of community composition at finer spatial scales. High initial similarity corresponds to low local variability. The relative influence of environmental selection and spatial autocorrelation was investigated through a variance partitioning approach involving pedo-climatic characteristics and land-use and spatial variables (geographic coordinates and Principal Coordinates of Neighbour Matrices; PCNM), respectively.

## Methods

### Soil samples

**Figure 1:**
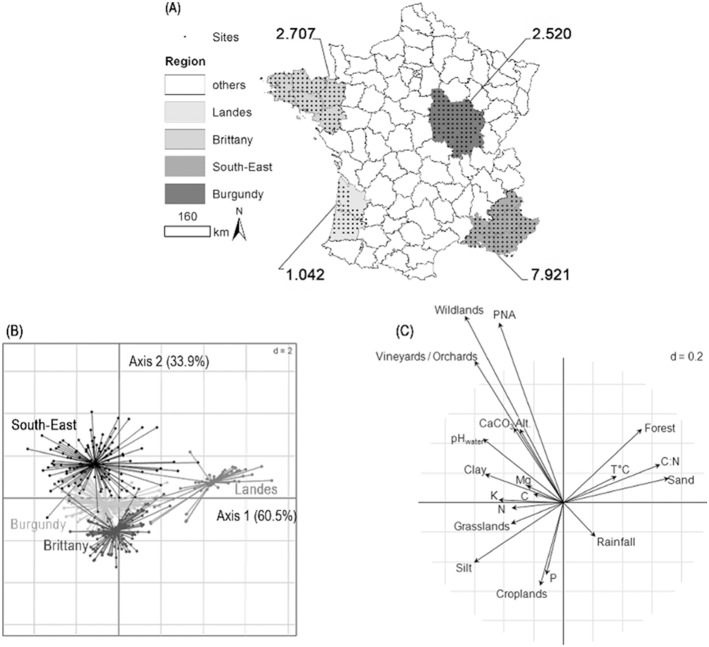
Comparison of the regions considered on the basis of their soil habitats. A. Soil average dissimilarity of soil habitat for the different regions (number linked to the corresponding region) and position of sites; B. Between group analysis of soil habitats according to the region; C. Correlation circle of the variables defining soil habitat in the between group analysis. The length of the arrow corresponds to the Pearson’s correlation coefficient for quantitative variables and to the correlation ratio for qualitative variables. Symbols: Alt.: Elevation; T°C: Sum of annual temperatures; P_ass_: Assimilable P; C:N: Carbon to Nitrogen ratio; C_org_: Organic Carbon content.

Soil samples were provided by the Soil Genetic Resource Conservatory (platform GenoSol, http://www.dijon.inra.fr/plateforme_genosol, [41]) and obtained from the soil storage facility of the RMQS (“Réseau de Mesures de la Qualité des Sols” = French Monitoring Network for Soil Quality). The RMQS database consists of observations of soil properties on a 16-km regular grid across the 550000 km^2^ French metropolitan territory and was designed to monitor soil properties [42]. The baseline survey consisting of 2,200 sites (each corresponding to a composite soil sample constituted of 25 soil cores) was completed in 2009. The sites were selected at the centre of each 16×16-km cell. In this study, we focused on a subset of 413 sites from the RMQS data set. The samples were organized into four regions: Brittany (131 sites), Burgundy (109 sites), Landes (52 sites) and South-East (121 sites, Fig. 1A) which are contrasted in terms of soil type, land-use (coarse level of the CORINE Land Cover classification; IFEN, http://www.ifen.fr; 7 classes: forest, crop systems, grasslands, particular natural ecosystems, vineyards/orchards, parkland and wild land), climate and geomorphology (Table S1). Within a region, sites were separated by 16 km at least. For each soil, the pedo-climatic characteristics considered were particle-size distribution, pH in water (pH_water_), organic carbon content (C_org_), N content, C:N ratio, soluble P contents, CaCO_3_ and exchangeable cations (Ca, K, Mg), sum of annual temperature (°C) and annual rainfall (mm). Physical and chemical analyses were performed by the Soil Analysis Laboratory of INRA (Arras, France) which is accredited for such analyses by the French Ministry of Agriculture.

### Bacterial and fungal community fingerprinting

#### Soil DNA extraction

For each soil sample, the equivalent of 1.5 g of dry soil was used for DNA extraction, following the procedure optimized by platform GenoSol [35]. Briefly, extraction buffer (100 mM Tris pH 8.0, 100 mM EDTA pH 8.0, 100 mM NaCl and 2% (w/v) SDS) was added to the sample in the proportion 3∶1 (v/w), with two grams of glass beads (106 µm diameter) and eight glass beads (2 mm diameter) in a bead-beater tube. All beads were acid washed and sterilized. The samples were homogenized for 30 s at 1600 rpm in a mini bead-beater cell disruptor (Mikro-dismembrator, S. B. Braun Biotech International), incubated for 30 min at 70°C in a water bath and centrifuged for 5 min at 7000 g and room temperature. The supernatant was collected, incubated on ice with 1/10 volume of 3 M potassium acetate (pH 5.5) and centrifuged for 5 min at 14000 g. DNA was precipitated with one volume of ice-cold isopropanol and centrifuged for 30 min at 13000 rpm. The DNA pellet was washed with ice-cold 70% ethanol and dissolved in 100 µl of ultra pure water. For purification, aliquots (100 µL) of crude DNA extracts were loaded onto PVPP (polyvinyl polypyrrolidone) minicolumns (BIORAD, Marne la Coquette, France) and centrifuged for 4 min at 1000 g and 10°C. This step was repeated if the eluate was opaque. The eluate was then collected and purified for residual impurities using the Geneclean Turbo kit as recommended by the manufacturer (Q Biogene, France).

#### PCR conditions

The bacterial ribosomal IGS was amplified using the PCR protocol described in Ranjard et al [43]. 12.5 ng of DNA was used as the template for PCR volumes of 25 µl. The fungal ribosomal ITS was amplified using the primer set ITS1F/ITS4-IRD800 (5′- CTTGGTCATTTAGAGGAAGTAA -3′/5′- IRD800-TCCTCCGCTTATTGATATGC -3′). 20 ng of DNA was used as the template for PCR volumes of 25 µl with the following PCR conditions: denaturation at 95°C for 3 min, 35 cycles of 30 s at 95°C, 45 s at 55°C and 1 min at 72°C, and a final elongation of 7 min at 72°C. The primer Tm was the same for bacterial IGS and fungal ITS. Every PCR products were purified using the MinElute Kit (QIAGEN, Courtaboeuf, France) and quantified using a calf thymus DNA standard curve.

#### ARISA fingerprinting conditions

2 µL of the PCR product was added to deionized formamide and denatured at 90°C for 2 min. Bacterial and Fungal ARISA fragments were resolved on 3.7% polyacrylamide gels under denaturing conditions as described in Ranjard et al [9] on a LiCor DNA sequencer (ScienceTec).

#### Image analysis

The data were analyzed using the 1D-Scan software (ScienceTec), converting fluorescence data into electrophoregrams, where peaks represented PCR fragments (100 to 110 peaks retained per sample, the resolution limit to avoid considering background noise). The height of the peaks was calculated in conjunction with the median filter option and the Gaussian integration in 1D-Scan, and represented the relative proportion of the fragments in the total products. Lengths (in base pairs) were calculated by using a size standard with bands ranging from 200 to 1659 bp. The data were then converted into a contingency table with prepRISA package in R.

### Statistical analyses

#### Characterization of habitat variability and average dissimilarity across regions

Habitats were compared between regions in a Hill & Smith multivariate analysis [44] using the ade4 package in R [45]–[46]. The analysis was applied to pedo-climatic characteristics, land-use and geomorphology, by centering and scaling the quantitative variables, and converting the qualitative ones into weighted binary variables (weight equal to 1/n; n is the number of classes for the qualitative variables). Differences between these regions were examined by between group analysis and tested by applying a Monte-Carlo permutation test (1000 permutations). The average dissimilarity between soil habitats was determined by transposing a method based on the dissimilarity matrix for communities [47] to soil habitat. The dissimilarity matrix for soil habitat was derived from the site coordinates in the Hill & Smith analysis, following equation 1 [9]:
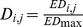
(1)Where D_i,j_ and ED_i,j_ are the dissimilarity and the Euclidean distance between sites i and j, respectively. ED_max_ is the maximum Euclidean distance observed between sites. The average dissimilarity between soil habitats was then calculated as follows [47]:
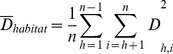
(2)

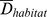
 is the average dissimilarity between soil habitats of soil habitat and n the number of sites in the region.

#### Evaluation of the similarity distance-decay relationship and initial similarity of Bacterial and Fungal community composition

The *similarity distance-decay* relationship was estimated as proposed by Harte et al [39] for organisms with large populations per taxa. From this relationship, the community composition turnover rates (z) for bacterial and fungal communities composition were derived as described in Ranjard *et al.* [9] following the method and the exponential model (equation 3) proposed by Harte et al [39]–[40] for microorganisms.
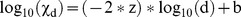
(3)Where χ_d_ is the observed Sørensen’s similarity between two soil samples that are d meters apart from each other; b is the intercept of the linear relationship and z the turnover rate of the community composition. The z estimate and its 95% confidence interval were derived from the slope (–2*z) of the relationship between similarity and distance by weighted linear regression. The overlap of the 95% confidence intervals was used to test for significant differences in community composition turnover rates between regions or between bacteria and fungi. The initial similarity of community composition was taken as the average similarity between sites 16 km apart and the 95% confidence intervals of the mean were determined [30].

#### Variance partitioning of community composition variations according to environmental filters and space

The relative importance of spatial variables, pedo-climatic characteristics and land-use in determining community composition turnover was tested by db-RDA [47]–[48]. Quantitative data were centered and scaled. Spatial variables were constructed from site coordinates (x, y, elevation) to reveal potential spatial trends at scales larger than the region, and of Principle Coordinates of Neighbour Matrices [49] in each region. The PCNM approach creates independent spatial descriptors that can be introduced in canonical analysis models to consider the spatial autocorrelation between sites in the model [49]. PCNMs with a significant Moran index (*P*<0.001) were selected. Land-use corresponded to the Corinne Land Cover classes recoded into dummy variables. Pedo-climatic characteristics consisted of climate and all the physico-chemical variables except sand. The most parsimonious model was obtained by forward selection from null to full model in two steps: a first step for selecting environmental variables and a second step for selecting the relevant PCNMs. Then, the pure effects of each set of filters or each individual filter were tested with an anova-like permutation test for canonical analyses (anova.cca function in vegan package, [50]). The PCNMs approach does not provide directly the range of the spatial descriptor. Nevertheless, Bellier et al [51] demonstrated that kriging approach could be applied to PCNMs to estimate their spatial range. Consequently, when PCNMs were selected in the most parsimonious model, their ranges were determined by standard kriging techniques (ordinary kriging with a Gaussian model). The hierarchy of these filters must nevertheless be considered with caution due to the small amounts of variance explained by each one. Land-use was not included in the filter-ranking since it corresponded to a set of categories and a global “land-use” category was already taken into account in the processes section. Maps of soil fungal community structure variations are provided as (Fig. S1, mapping methodology is described in the legend).

## Results

### Heterogeneity of soil habitat

The four regions were selected for their contrasting environmental heterogeneity as demonstrated by the between group analysis (Fig. 1B) and comparison of the calculated average dissimilarity between soil habitats (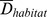) (Fig. 1A and C). Multivariate analysis revealed a clear discrimination of the four regions on the first and second axes (Monte-Carlo permutation test, P<0.001): Landes was significantly discriminated from Brittany, Burgundy and South-east on the first axis and these three regions were discriminated from each other on the second axis. In addition, the environmental variability strongly differed between the four regions as demonstrated by the dispersal of sites in the factorial map. Sites from Landes were less dispersed on the factorial map than the sites from Brittany or Burgundy, which were less dispersed than sites from South-East. The calculated 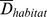 ranged from 1.042 in the Landes to 7.921 in the South-East, with intermediate values for Burgundy and Brittany (2.520 and 2.707, respectively; Fig. 1A). It provided the same discrimination between regions. Fig. 1C shows that the four regions could mainly be distinguished according to land-use (*e.g.*: 86% of the Landes sites are forest sites), a restricted set of soil physico-chemical characteristics (sand and silt contents, pH_water_ and CaCO_3_ content, assimilable P content and organic matter quality as measured by C:N ratio) and by differences in elevation. Climatic conditions did not play a significant role in regional discrimination.

### Distance-Decay Relationship for bacterial and fungal soil communities

**Figure 2:**
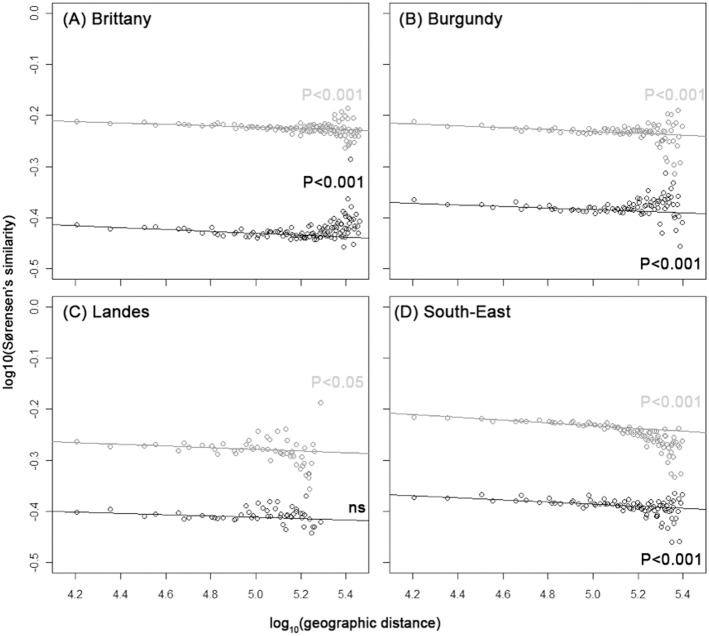
Distance-Decay Relationships for bacteria and fungi. Each panel correspond to a region: Brittany (A), Burgundy (B), Landes (C) and South-East (D) Points the average Sørensen’s similarity between sites for each distance class. Lines represent the regression model based on the whole set of paired comparisons; for bacteria (grey) and fungi (black). The equations for the regression models were as follows: (**A**) Brittany: Bacteria***: log10(Sørensen’s similarity) = −0.014×log10(geographic distance)−0.156; Fungi***: log10(Sørensen’s similarity) = −0.017×log10(geographic distance)−0.350; (**B**) Burgundy: Bacteria*** log10(Sørensen’s similarity) = −0.018×log10(geographic distance)−0.144; Fungi***: log10(Sørensen’s similarity) = −0.015×log10(geographic distance)−0.316; (**C**) Landes: Bacteria*: log10(Sørensen’s similarity) = −0.017×log10(geographic distance)−0.198; Fungi ^ns^: log10(Sørensen’s similarity) = −0.012×log10(geographic distance)−0.357; (**D**) South-East: Bacteria***: log10(Sørensen’s similarity) = −0.027×log10(geographic distance)−0.101; Fungi***: log10(Sørensen’s similarity) = −0.019×log10(geographic distance)−0.298. A graph with points representing all paired-comparisions between sites as points can be found in Figure S2. Significance of the model is indicated as an exponent for each organism: ns: not significant; P<0.05: *; P<0.01: **, P<0.001: ***.

Bacterial and fungal community similarity decreased with increasing distance in each region (Fig. 2). Community similarity was systematically higher for bacteria than for fungi in all regions at small or large distances. For bacteria, the linear regression model was highly significant in each region (*P*<0.001, Fig. 2) except in Landes where it was just below the significance threshold of 5% (*P*<0.02). For fungi, the linear regression model was highly significant in each region (P<0.001) except Landes (Fig. 2).

Community composition turnover rates were derived from the parameters of the linear regression. The community composition turnover rates for the bacterial and fungal communities ranged from 0.006 to 0.013 (Table 1). No significant differences were highlighted between these organisms when the community composition turnover rates were compared within each region. When the community composition turnover of bacterial or fungal communities was compared between regions, a significant difference was only found between Brittany and the South-East (P<0.05) for bacteria.

**Table 1:** Regression parameters of the Distance-Decay Relationships for Bacteria and Fungi.

Region	Parameter	Organism	Estimate	95% Confidence interval
Brittany (131)	Z	Bacteria	0.007	[0.005; 0.009]
		Fungi	0.009	[0.006; 0.011]
	Initial similarity	Bacteria	61.4%	[60.4%; 62.3%]
		Fungi	38.5%	[37.7%; 39.4%]
Burgundy (109)	z	Bacteria	0.009	[0.006; 0.012]
		Fungi	0.008	[0.003; 0.012]
	Initial similarity	Bacteria	61.4%	[60.4%; 62.5%]
		Fungi	43.1%	[42.0%; 44.3%]
Landes (52)	z	Bacteria	0.009	[0.001; 0.016]
		Fungi	0.006	[−0.001; 0.013]
	Initial similarity	Bacteria	54.4%	[52.9%; 56.0%]
		Fungi	39.7%	[38.4%; 40.9%]
South-East (121)	z	Bacteria	0.013	[0.011; 0.016]
		Fungi	0.009	[0.006; 0.013]
	Initial similarity	Bacteria	60.9%	[59.9%; 61.9%]
		Fungi	42.4%	[41.5%; 43.3%]

The number of observations per region is provided in brackets beside the name of the region. The community composition turnover rate (z) and the initial similarity are derived from the slope of the regression (−2z) and the mean of similarity at 16 km; respectively. The statistical comparison between region and organism was performed by examining the overlap of the 95% confidence intervals of turnover rates or initial similarities.

The initial similarity was always higher for bacterial communities than for fungal communities within each region, ranging respectively from 54.4% to 61.4% and from 39.7% to 43.1%. The initial similarity of the bacterial community in Landes was significantly lower than in the other regions, which did not differ from each other. For fungi, the initial similarities in Landes and Brittany were similar and significantly lower than those in Burgundy and the South-East.

These results were confirmed by a covariance analysis comparing the models between organisms within a region and between regions for a given organism (data not shown).

### Variance partitioning of community composition variations

**Figure 3:**
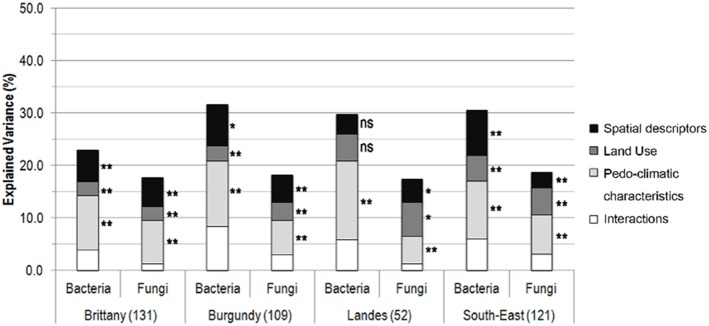
Variance partitioning of bacterial and fungal community composition. The number indicated in brackets corresponds to the number of samples for the region. Significance levels: ns: not significant; P<0.05: *; P<0.01: **, P<0.001: ***.

The relative importance of the sets of spatial variables, land-use and pedo-climatic characteristics on variations in bacterial and fungal community composition was tested by db-RDA using the Sørensen index (Fig. 3). The amount of variance in bacterial and fungal community composition explained by the three sets of filters ranged from 17% to 32%. The significance of the interactions between the three sets of filters could not be tested but always explained a small amount of the total variance (from 1.2% to 8.4%).

Among the sets of filters, soil pedo-climatic characteristics were the main contributor to variations in bacterial and fungal community composition. Spatial variables systematically explained a lower amount of variance than pedo-climatic characteristics, but a higher amount than land-use for both bacteria and fungi except for bacteria in Landes region where neither spatial variables nor land-use were significant.

The amount of variance explained by pedo-climatic characteristics for bacteria or fungi was always significant, ranging from 5% to 15%, and was similar between regions. Nevertheless, within each region, the amount of variance explained by soil pedo-climatic characteristics was always higher for bacteria than for fungi (Fig. 3).

Spatial variables significantly explained part of the community composition variations in all regions except for bacteria in Landes region. When significant, spatial variables represented from 3% to 9% of the total variance and were of the same order of magnitude both between bacteria and fungi and between regions (Fig. 3).

Similarly, land-use explained a significant amount of community composition variations in all regions except for bacteria in Landes region. The amount of explained variance ranged from 2.6% to 6.5% of the total variance. Within each region, land-use explained similar amounts of bacterial and fungal community variations when significant. Between regions, amounts of variance explained by land-use were similar for both bacteria and fungi (Fig. 3).

### Hierarchy of environmental filters

**Figure 4:**
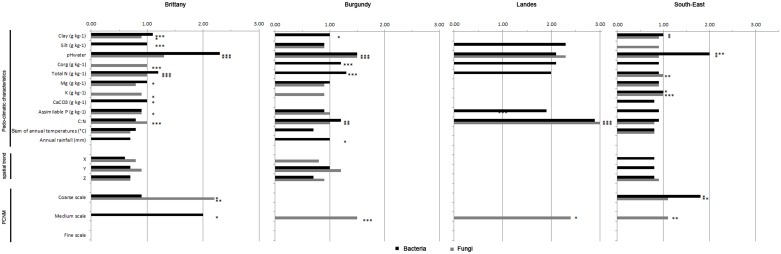
Variations of microbial communities partitionned according to edaphic variables and space. For each organism and region, only variables retained in the most parsimonious model are presented and their pure effect is tested by a permutation test. Significance levels are: P<0.05: *; P<0.01: **, P<0.001: ***. Missing values or variables indicate that the variable was not retained in the model. Sand was removed prior to model evaluation since it was represented by the opposite of the sum of silt and clay content. Rainfall: Sum of annual rainfall (mm). Temperature: Sum of annual temperature (°C). Spatial components were summarized according to the scale considered: trend (x, y and z coordinates), coarse, medium or fine. The interval in brackets indicates the numbers of PCNMs retained in the model for each scale. The proportion of variance for each scale was determined as the sum of the pure effects of each PCNM when these were significant. Coarse, medium and fine scales correspond to PCNM with a spatial range of 80 to 120 km, 40 to 65 km and less than 40 km; respectively.

Within the sets of soil pedo-climatic characteristics and spatial variables, the pure effects of individual filters on bacterial and fungal community structure are presented in Figure 4. These pure effects account for relatively small proportions of the total variance (from 0.7% to 6.5%) because of the large number of filters explaining the total variance of bacterial or fungal community compositions. Regarding the pedo-climatic characteristics, the significant filters for soil bacterial communities in Brittany were first pH and secondly N content. For fungi, these were the quality of organic matter resource as indicated by the selection of N content, C_org_ content and C:N ratio. The least significant filters corresponded to soil texture and other soil nutrients for bacteria (clay and silt contents, Mg and CaCO_3_ concentrations) and fungi (clay content, K and P concentrations, Figure 4). In Burgundy, as in Brittany, the soil bacterial and fungal communities were principally affected by pH. Beside pH, bacterial community composition was shaped by the quality of organic matter resource (N content, C_org_ content, C:N ratio) followed by clay content and annual rainfall. The fungal community composition was shaped by C:N ratio only. The only filter that had a significant effect in the Landes region, on both soil bacteria and soil fungi, was the C:N ratio. The pH was the most important filter, followed by clay content and K concentration, for bacteria in the South-East. In this region, N content and K concentration were more important filters for fungi than pH and clay content.

The spatial variables corresponded to the sites coordinates and 16 significant PCNM eigenfunctions, each representing a different spatial scale of analysis (coarse, medium and fine scales, Figure 4). Longitude, latitude or altitude coordinates did not influence community composition except latitude for fungi in Burgundy region (Figure 4). PCNMs representing spatial structures of 80–120 km radius explained significant amounts of variance in the composition of bacterial and fungal communities in Brittany and South-East. This highlighted that these communities were spatially structured at a coarse spatial scale. Similarly, PCNMs representing spatial structures of 40–65 km radius explained significant amounts of variation in the composition of bacterial community in Brittany and of fungal community in Burgundy, South-East and Landes. This showed the spatial structuration of these communities at medium spatial scales. PCNMs accounting for fine scale variables were neither significant for bacteria nor fungi in any region.

## Discussion

The four regions were selected to challenge the hypothesis that different levels of environmental heterogeneity, *i.e.* different habitat diversity and fragmentation for soil bacteria and fungi, results in different community composition turnovers [9], [14] and to compare their determinism. The multivariate analysis and the calculated 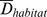 both highlighted a significant gradient in environmental heterogeneity following the sequence: Landes<Burgundy≤Brittany<<South-East. The four regions were mainly discriminated by environmental variables already demonstrated to influence soil microbial community abundance and diversity, such as land-use [52] and soil characteristics (texture, pH_water_, P content and C:N ratio; [6], [14], [33], [35]). Among these four regions, Landes and South-East represent two extremes of environmental heterogeneity. Landes was distinct in having sites with conifer forest on acidic soils and low altitudinal variations. This specificity may turn Landes into an outlier for its environmental variability. On the other hand, South-East is characterized by a strong altitudinal gradient and a mosaic of land-use. In comparison, Burgundy and Brittany were also characterized by a mosaic of land-use but were more marked by croplands than South-East and less by forests. In addition, these two regions presented intermediate soil types regarding Landes and South-East: silty-clay calcic soils and silty acidic soils, respectively; associated to intermediate levels of organic Carbon content. Altogether, these observations highlight that these four regions allow the consideration of a large range of environmental conditions associated to different levels of environmental heterogeneity. Therefore, community similarity turnover rates can be confronted to environmental heterogeneity and their comparison may lead to a consensus regarding the environmental filters shaping soil microbial communities.

In all regions, except Landes, the soil bacterial and fungal communities were spatially structured as indicated by the significant DDR. This supports that the concept of DDR for soil microorganisms may be generalized, as suggested by several other studies [1], [9], [12], [24], [53]. The estimated bacterial and fungal community composition turnover rates ranged from 0.006 to 0.013. This is in agreement with the recent community composition turnover rates observed for soil microorganisms [10], [14], [24]. However, the turnover rates estimates were low. This could be due to technical limitations; particularly the low taxonomic resolution of DNA fingerprinting. Species variations were aggregated by the DNA fingerprints into few dominant bands, which precluded the accumulation of new minor species with increasing distance [54]–[55]. This would be supported by the higher community composition turnovers observed in Zinger [24] with the high resolution level provided be the pyrosequencing approach. The sampling design could also have led to low estimates of the community composition turnover rate. Indeed, lower composition turnover rates are commonly observed in regions composed of contiguous habitats [13] with gradual variations of habitat characteristics across sites and higher rates of community composition turnover have been observed at finer taxonomic levels and spatial scales [56].

Beside these technical points, community composition turnover rates were very close within each region for bacteria and fungi. This observation was in agreement with maps of bacterial and fungal communities’ structures which revealed large patches of *ca.* 100–140 km radius (see Fig. 2 in [7] for bacteria and Fig. S1 for fungi). Altogether, this indicated a low-level of aggregation of both communities on a broad spatial scale [57]. This observation would suggest that differences in terms of biological and ecological features (habitat characteristics, colonization modes, trophic requirements, biotic interaction, dormancy; [6], [58]–[59]) would not lead to different rates of community composition turnover on a broad spatial scale. This would be supported by strong biotic interactions between bacterial and fungal communities highlithed by a significant correlation between bacterial and fungal communities’ compositions. Nevertheless, this correlation was much lower than that between each community composition and environmental variables (data not shown). This strong dependency to environmental conditions resulted in a trend towards higher turnover rates in regions with higher 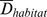. This would corroborate our first hypothesis that the microbial DDR is positively related to environmental heterogeneity. This observation was more marked and significant for bacterial communities but only a tendency for fungal communities. Although this conclusion is based on analyses of only 4 regions, it is in agreement with Ranjard *et al.* [9] who demonstrated a correlation between habitat heterogeneity and turnover rate for soil bacterial communities on a broad spatial scale, and with other microbial and macrobial studies [24], [30]–[31], [60].

The differences between the sensitivities of soil bacteria and fungi to environmental heterogeneity could be due to the ecology of soil fungi [58] at the spatial scale of this study. Nevertheless, according to Peay et al [25]–[26], ectomycorhizal fungi are spatially structured at very fine spatial scales. This would suggest that these organisms are strongly dependent on environmental conditions but that the grain size of this study (252 km^2^) did not allow perceiving this dependence. This hypothesis would be supported by the estimated similarity of the microbial communities at a local spatial scale (initial similarity, [30]). Indeed, the initial similarity was systematically higher for bacterial than for fungal communities. According to Morlon et al [57], this would indicate that fungal communities are more aggregated than bacterial communities, *i.e.* more variable than bacterial communities, at a local spatial scale. This would lead to the conclusion that fungi are somehow more dependent on environmental conditions than bacteria at a local scale, in agreement with Peay et al [25]–[26] and the numerous biological interactions they are involved in.

The observed patterns and significant turnover rates result from different ecological processes [8] which may shape differently bacterial and fungal community composition on a broad spatial scale according to their relative importance. Recent studies have demonstrated that the main processes involved in increasing or decreasing community composition turnover rate are environmental selection and dispersal, respectively [8]–[9]. Dispersal is commonly supposed, but not empirically demonstrated to be high for microbes [61]. The maintenance of significant turnover rates, as recorded in our study, thus requires environmental selection to be high and dispersal not infinite. This hypothesis was tested by partitioning the β-diversity variations of microbial communities within regions. This partition was made according to filters involved in environmental selection: soil pedo-climatic characteristics and land-use; and to spatial variables characterizing spatial autocorrelation unexplained by the environmental variables. The residual spatial autocorrelation could result from dispersal limitations, but also from unmeasured spatially autocorrelated environmental variables despite the extensive number of environmental variables considered in this study. The amount of explained variance ranged from 23% to 32% for bacteria and from 17.5% to 18.8% for fungi. These values are within the range reported in the literature for the whole communities of bacteria [8], [14] and fungi [25]–[26], [62].

Soil characteristics accounted for higher amounts of variance, for both bacteria and fungi, than spatial variables. This supports the idea that soil microbial communities are primarily affected by environmental selection and secondly by other processes leading to spatial structuration independently of the environment [2], [7]–[8], [38]. More precisely, environmental selection was mainly driven by pedo-climatic characteristics, which accounted for larger amounts of variance than land-use. This is in agreement with several studies conducted on a broad spatial scale evidencing the higher dependency of bacterial and fungal communities on soil physico-chemical characteristics [18], [38], [63] than on land-use. Nevertheless, the amount of variance explained by pedo-climatic characteristics was higher for bacteria than for fungi, highlighting the greater effect of soil habitat on shaping the bacterial community. This observation is supported by the higher diversity and reactivity of the soil bacterial community to changes in surrounding conditions which, in turn, leads to a community structure that is better fitted to the habitat characteristics [13], [58]. On the contrary, land-use explained a higher amount of fungal community variance than of bacterial community variance. This latter could be due to plant-soil microbe interactions resulting from the type of vegetative cover [19], [38], [52], [64] as well as from human activities, especially agricultural or industrial practices [35]; [37] potentially affecting fungi more strongly than bacteria. This difference would be in agreement with a highly patchy distribution of soil fungi at a local scale as suggested above by the low level of initial similarity. Altogether, these results support the postulate of Baas-Becking “the environment selects” in summarizing microbial biogeography.

Nevertheless, variance partitioning also highlighted that bacterial and fungal communities are spatially structured independently of environmental characteristics since spatial variables accounted for a significant amount of community variance. A hypothesis in explaining this spatial autocorrelation is that soil bacterial and fungal communities might be dispersal limited, even if this result may also be related to unmeasured, spatially structured, environmental variables. This hypothesis would be supported by the study of Ranjard et al [9] demonstrating significant turnover rates of bacterial diversity in fully homogenous regions and by the consideration of several soil physico-chemical characteristics in the variance partitioning approach of the present study. Nevertheless, this must be confirmed by more in-depth studies since few evidence remains to date on the shaping of bacterial and fungal communities by limited dispersal [8], [17], [26]–[27], [65]. Comparisons have indicated that fungi tend to be less dependent on spatial variables than bacteria. This result is surprising since fungi are demonstrated to be dispersal limited [27] and bacteria are generally expected to disperse over larger distances, even wider than our regional scale [14], [66]. It was estimated in the literature that 10^18^ viable bacteria were transported annually in the atmosphere between continents [61]. Moreover, particular fungal populations have been demonstrated to disperse over short distances, *e.g.* ectomycorrhiza at the “plant island scale” [25]–[26], or to be endemic [27]. On the other hand, variance explained by spatial variables was similar in each region for fungi, whereas it followed the environmental heterogeneity gradient: Landes<Brittany≤Burgundy<South-East for bacteria. This observation supports the hypothesis that bacteria are more sensitive to geomorphology and that the presence of natural barriers (*e.g.* mountains), or a higher fragmentation of landscape in the South-East, due to a mosaic of agricultural and natural plots [9] may lead to significant spatial variations.

Our last objective was to identify and rank the environmental filters and the spatial variables involved in environmental selection process and spatial autocorrelation of bacteria and fungi. To do so, the environmental and spatial filters selected in the variance partitioning approach were ranked according to their pure effect on bacterial and fungal community composition turnover. First, environmental selection was mainly based on soil characteristics but not on climatic conditions. This is in accordance with previous studies highlighting the weak influence of climate on soil microbial diversity on a broad spatial scale [6]–[7], [35], [37], [67], [68]. Nevertheless, recent studies also demonstrated strong differences between bacterial communities across different biomes, especially cold deserts *versus* temperate biomes and hot deserts, suggesting that climate may play a role [32] at very large spatial scales. Overall, the main soil characteristics identified as important filters for soil bacterial and fungal community composition were pH, trophic resources (N, C, K, P contents; C:N ratio) and texture (clay, silt content). These findings are consistent with the literature where i) pH is regularly identified as the main filter for both soil bacteria [6], [16]–[17] and fungi [25], [62], ii) soil organic C and N contents and the C:N ratio constitute important components of microbial niches [18], [32], [35], [58], and iii) texture determines the size and stability of soil micro-habitats [18], [69], [70]. The hierarchy of these filters depended on the type of organisms and the regions. The above-described hierarchical sequence (pH>trophic resources≥texture) was observed for three of the four regions, but not for Landes. In the Landes region, the filter variability is low and environmental selection is basically driven by organic matter quality (C:N ratio). This is related to the large number of conifer forests sites leading to soil organic matter with a high C:N ratio and strong recalcitrance to microbial decomposition. A strong selection of particular populations with enzymatic ability to transform this organic matter is occurring in such soils [58], which is deeply influencing the corresponding community structure. Comparison of the overall filter hierarchies for bacteria and fungi in a given region did not reveal any discrepancy for the primary drivers. The only differences were observed for secondary filters, including C:N ratio and mineral nutrients (such as K and P) for fungi, and clay and N content for bacteria. This is consistent with the well-known dependency of the soil fungal community on soil P content [34] and soil organic matter quality [58]. As regards bacteria, clay content is positively correlated with the biotic capacity of soil as well as its indigenous bacterial diversity by enhancing the level of protection of the soil habitat and the retention of nutrients [69]–[70]. Second, regarding spatial autocorrelation, different scales were derived from the hierarchy of spatial variables and their range. The main scale identified was the coarse scale (80 to 120 km radius), for both bacteria and fungi in Brittany and South-east regions. This spatial scale is smaller than the one at which soil habitat changed on the RMQS Network (150 to 470 km, [9]), whereas it is in agreement with the large patches obtained by mapping the bacterial and fungal community structure over these regions [7] and Fig. S1). In addition, finer spatial scales (medium scales; 40 to 65 km radius) were also identified as significant in Burgundy, Landes and South-East for fungi and in Brittany for bacteria. Altogether, this scale dependency would support a hypothesis for the dispersal limitation of bacterial and fungal communities [33]. This scale dependency is in agreement with Martiny et al [14] for bacteria and is supported by the observations of Peay et al [25]–[26] on soil fungi. As in other studies at continental scales [14], this highlights the importance of considering multiple scales to better understand microbial ecology.

Altogether, our study demonstrated the spatial structuring of soil bacterial and fungal communities on local to coarse scales, which was based on environmental selection and on an unexplained spatial autocorrelation that could be related to limited dispersal. Selection and spatial autocorrelation were shown to have a similar influence on soil bacteria and soil fungi but the filters involved could differ depending on the environmental heterogeneity. Nevertheless, the comparison of bacterial and fungal communities helped to propose a primary consensus regarding the environmental filters shaping soil microbial community composition as a whole: Land-Use and pH are the primary filters, followed by trophic resources quantity (organic carbon content and nitrogen content) and then quality (C:N ratio). The results of this study increase our knowledge on the effects of soil habitat and provide insights in the scale at which dispersal may occur according to the ecological attributes of bacteria and fungi. However further investigations, based on up-scaling approaches, are now required to: i) provide a direct measurement of bacterial cells dispersal which is now crucial to demonstrate limited dispersal of bacteria; ii) identify the filters operating at each spatial scale. Especially, these upscaling approaches could be associated to high-throughput sequencing to achieve a finer resolution on the communities. This would improve our ability to sustainably manage soil biodiversity.

## Supporting Information

Figure S1 **Maps of interpolated MULTISPATI scores for the first three MULTISPATI axes (columns) and for the four geographical regions (rows).** Each map was generated as described in Dequiedt et al (2009) and corresponds to the spatial synthesis of the F-ARISA genetic structure of indigenous fungal communities from the corresponding soils sampled in the four regions of France. Colours on the map are proportional to the score of each soil sample on each MULTISPATI axis following the scale provided at the bottom of the figure. Below each column, the empirical variogram is provided for each MULTISPATI axis.
(DOC)

Figure S2 **Distance-Decay Relationship for bacteria and fungi.** Each panel correspond to: **(A–D): Bacteria** in Brittany, Burgundy, Landes and South-East; **(E–H): Fungi** in Brittany, Burgundy, Landes and South-East. Points represent paired-comparisons between sites and line the linear model. The equations for the regression models were as follows: **(A)** log10(Sørensen’s similarity) = −0.014×log10(geographic distance)−0.156; **(B)** log10(Sørensen’s similarity) = −0.018×log10(geographic distance)−0.144; **(C)** log10(Sørensen’s similarity) = −0.017×log10(geographic distance)−0.198; **(D)** (Sørensen’s similarity) = −0.027×log10(geographic distance)−0.101; **(E)** log10(Sørensen’s similarity) = −0.017×log10(geographic distance)−0.350; **(F)** log10(Sørensen’s similarity) = −0.015×log10(geographic distance)−0.316; **(G)** log10(Sørensen’s similarity) = −0.012×log10(geographic distance)−0.357; **(H)** log10(Sørensen’s similarity) = −0.019×log10(geographic distance)−0.298. Significance of the model is indicated as an exponent for each organism: ns: not significant; P<0.05: *; P<0.01: **, P<0.001: ***.
(DOCX)

Table S1 **Summary statistics of regions characteristics.** PNE: Particular Natural Ecosystems, SE: standard error of the mean.
(DOC)
