# Supplementary material for: Similar Processes but Different Environmental Filters for Soil Bacterial and Fungal Community Composition Turnover on a Broad Spatial Scale
Source: PLoS One. 2014 Nov 3;9(11):e111667. doi: 10.1371/journal.pone.0111667 (PMC4218796; doi:10.1371/journal.pone.0111667)
Supplement: Figure S1 — Maps of interpolated MULTISPATI scores for the first three MULTISPATI axes (columns) and for the four geographical regions (rows). Each map was generated as described in Dequiedt et al (2009) and corresponds to the spatial synthesis of the F-ARISA genetic structure of indigenous fungal communities from the corresponding soils sampled in the four regions of France. Colours on the map are proportional to the score of each soil sample on each MULTISPATI axis following the scale provided at the bottom of the figure. Below each column, the empirical variogram is provided for each MULTISPATI axis. (DOC) [file pone.0111667.s001.doc]

**Supplementary Information**


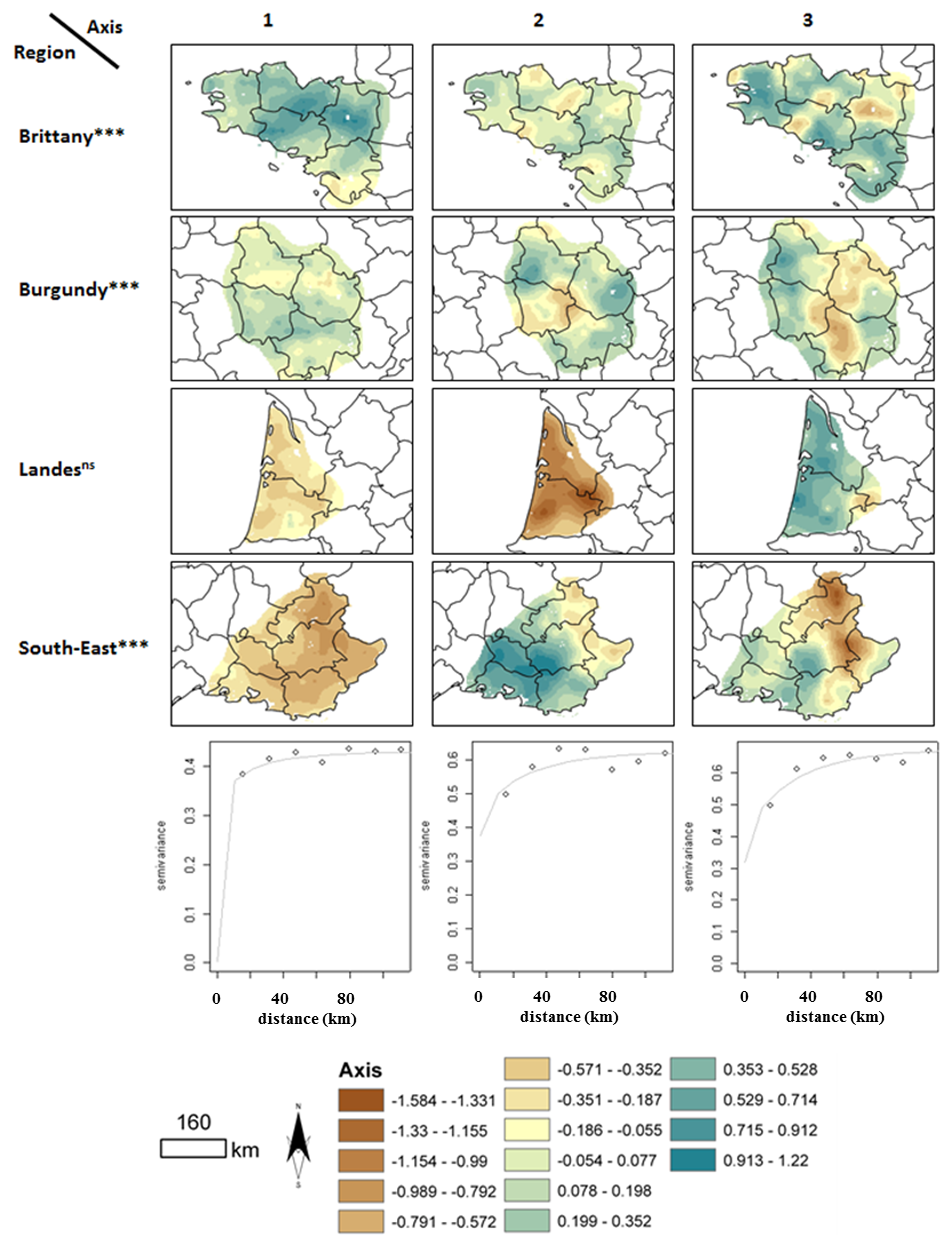


**Figure S1. Maps of interpolated MULTISPATI scores for the first three MULTISPATI axes (columns) and for the four geographical regions (rows).** Each map was generated as described in Dequiedt et al (2009) and corresponds to the spatial synthesis of the F-ARISA genetic structure of indigenous fungal communities from the corresponding soils sampled in the four regions of France. Colours on the map are proportional to the score of each soil sample on each MULTISPATI axis following the scale provided at the bottom of the figure. Below each column, the empirical variogram is provided for each MULTISPATI axis.

Mapping of soil fungal community variations in the four French regions considered in the study. Maps were obtained by standard kriging of the 3 first axes of a multivariate spatial analysis (MULTISPATI) on bands relative abundance data. As described in Dray et al. (2008), MULTISPATI introduces a spatial weighting matrix in a multivariate analysis (here a principal components analysis), allowing to maximize spatial autocorrelation in the sample. The spatial weighting matrix is derived from a connectivity matrix based on sites coordinates (longitude, latitude) and on a neighborhood relationship corresponding here to the one-square move of the Queen on a chess board.

Because the MULTISPATI analysis is a multivariate analysis and because more than one principle component (axis) was relevant to examine the spatial variations of the fungal community, the three first axes were mapped in the four regions. In these maps, color differences highlight community changes. For example, on axis 1, the Landes regions experiment low color variations regarding other regions. This suggests that fungal community are less spatially structured in Landes than in the other regions.
