# Supplementary material for: Similar Processes but Different Environmental Filters for Soil Bacterial and Fungal Community Composition Turnover on a Broad Spatial Scale
Source: PLoS One. 2014 Nov 3;9(11):e111667. doi: 10.1371/journal.pone.0111667 (PMC4218796; doi:10.1371/journal.pone.0111667)
Supplement: Figure S2 — Distance-Decay Relationship for bacteria and fungi. Each panel correspond to: (A–D): Bacteria in Brittany, Burgundy, Landes and South-East; (E–H): Fungi in Brittany, Burgundy, Landes and South-East. Points represent paired-comparisons between sites and line the linear model. The equations for the regression models were as follows: (A) log10(Sørensen’s similarity) = −0.014×log10(geographic distance)−0.156; (B) log10(Sørensen’s similarity) = −0.018×log10(geographic distance)−0.144; (C) log10(Sørensen’s similarity) = −0.017×log10(geographic distance)−0.198; (D) (Sørensen’s similarity) = −0.027×log10(geographic distance)−0.101; (E) log10(Sørensen’s similarity) = −0.017×log10(geographic distance)−0.350; (F) log10(Sørensen’s similarity) = −0.015×log10(geographic distance)−0.316; (G) log10(Sørensen’s similarity) = −0.012×log10(geographic distance)−0.357; (H) log10(Sørensen’s similarity) = −0.019×log10(geographic distance)−0.298. Significance of the model is indicated as an exponent for each organism: ns: not significant; P<0.05: *; P<0.01: **, P<0.001: ***. (DOCX) [file pone.0111667.s002.docx]

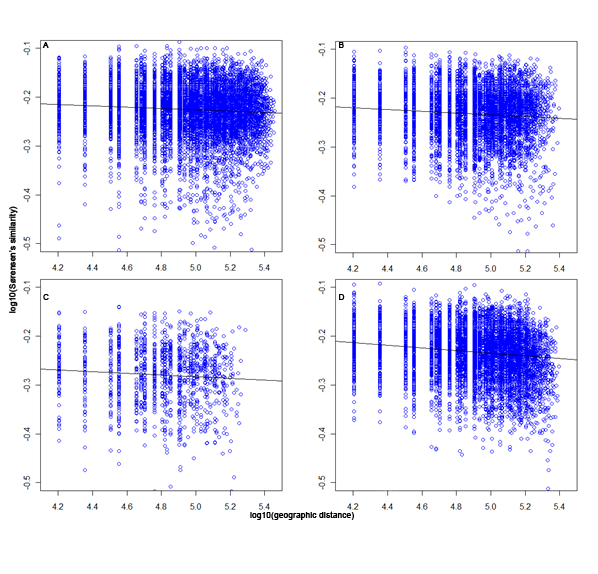

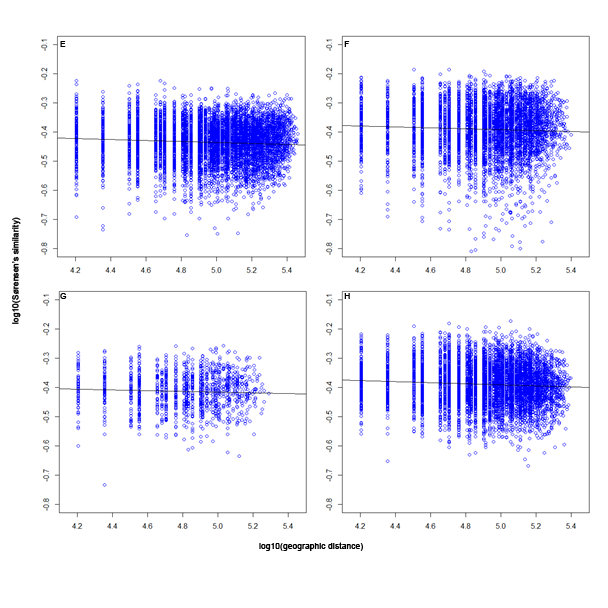


**Figure S2. Distance-Decay Relationship for bacteria and fungi.** Each panel correspond to: **(A – D): Bacteria** in Brittany, Burgundy, Landes and South-East; **(E – H): Fungi** in Brittany, Burgundy, Landes and South-East. Points represent paired-comparisons between sites and line the linear model. The equations for the regression models were as follows: **(A)** log10(Sørensen’s similarity) = -0.014 x log10(geographic distance) - 0.156; **(B)** log10(Sørensen’s similarity) = -0.018 x log10(geographic distance) - 0.144; **(C)** log10(Sørensen’s similarity) = -0.017 x log10(geographic distance) - 0.198; **(D)** (Sørensen’s similarity) = -0.027 x log10(geographic distance) - 0.101; **(E)** log10(Sørensen’s similarity) = -0.017 x log10(geographic distance) - 0.350; **(F)** log10(Sørensen’s similarity) = -0.015 x log10(geographic distance) - 0.316; **(G)** log10(Sørensen’s similarity) = -0.012 x log10(geographic distance) - 0.357; **(H)** log10(Sørensen’s similarity) = -0.019 x log10(geographic distance) - 0.298. Significance of the model is indicated as an exponent for each organism: ns: not significant; P<0.05: *; P<0.01: **, P<0.001: ***
