# Supplementary material for: Similar Processes but Different Environmental Filters for Soil Bacterial and Fungal Community Composition Turnover on a Broad Spatial Scale
Source: PLoS One. 2014 Nov 3;9(11):e111667. doi: 10.1371/journal.pone.0111667 (PMC4218796; doi:10.1371/journal.pone.0111667)
Supplement: Table S1 — Summary statistics of regions characteristics. PNE: Particular Natural Ecosystems, SE: standard error of the mean. (DOC) [file pone.0111667.s003.doc]

**Supplementary information**

|  |  | | **Brittany**  **n=133** | **Burgundy**  **n=109** | **Landes**  **n=52** | **South-East**  **n=121** |
| --- | --- | --- | --- | --- | --- | --- |
|  | Altitude (m) | Mean (SE)  Median  [min; max]  CV (%) | 82.9 (5.5)  69  [0.0; 282.0]  76 | 286.6 (10.6)  260  [84.0; 707.0]  39 | 56.9 (5.1)  51  [0.0; 147.0]  64 | 794.0 (64.9)  594  [0.0; 2759.0]  90 |
| Climate | Annual rainfall (mm) | Mean (SE)  Median  [min; max]  CV (%) | 971.4 (14.0)  923.9  [707.3; 1363.2]  17 | 908.4 (11.6)  888.4  [735.9; 1361.7]  13 | 999.0 (18.1)  959.6  [803.4; 1368.4]  13 | 861.1 (15.4)  867.9  [535.7;1246.1]  20 |
|  | Sum of annual temperatures (°C) | Mean (SE)  Median  [min; max]  CV (%) | 142.7 (0.5)  141.9  [128.6; 161.4]  4 | 133.2 (0.5)  134.5  [121.4; 145.6]  4 | 164.4 (0.5)  164.1  [156.9; 172.8]  2 | 146.4 (2.9)  154.1  [68.5; 194.5]  20 |

| Land use | Croplands  Forests  Grasslands  Vineyards/Orchard  PNE  Parks  Wild lands | Number of observations | 92  10  30  1  -  -  - | 40  33  35  1  -  -  - | 5  45  -  -  1  1  - | 22  48  24  11  13  -  3 |
| --- | --- | --- | --- | --- | --- | --- |
| Soil physico-chemical caracteristics | Sand (g kg^-1^) | Mean (SE)  Median  [min; max]  CV (%) | 321.2 (13.9)  728  [9.0; 801.0]  50 | 255.5 (18.7)  197  [12.0; 818.0]  76 | 891.0 (20.4)  943  [320.0; 986.0]  16 | 316.1 (18.2)  246  [18.0; 913.0]  63 |
|  | Silt (g kg^-1^) | Mean (SE)  Median  [min; max]  CV (%) | 493.9 (12.0)  515  [140.0; 738.0]  28 | 424.2 (12.8)  419  [104.0; 765.0]  31 | 57.9 (12.0)  28  [2.0; 446.0]  148 | 372.1 (10.4)  377  [54.0; 651.0]  31 |
|  | Clay (g kg^-1^) | Mean (SE)  Median  [min; max]  CV (%) | 185.0 (7.3)  171  [59.0; 734.0]  45 | 320.3 (14.7)  282  [78.0; 733.0]  48 | 51.1 (9.7)  27.5  [5.0; 402.0]  136 | 311.8 (12.3)  306  [33.0; 707.0]  43 |
|  | pH_water_ | Mean (SE)  Median  [min; max]  CV (%) | 5.8 (0.1)  5.8  [4.0; 8.2]  12 | 6.4 (0.1)  6.4  [4.2; 8.3]  19 | 4.9 (0.1)  4.6  [4; 7.5]  15 | 7.7 (0.1)  8.1  [4.9; 8.9]  13 |
|  | C_org_ (g kg^-1^) | Mean (SE)  Median  [min; max]  CV (%) | 25.5 (1.1)  23.1  [6.0; 104.3]  47 | 28.8 (1.9)  23.0  [8.1; 104.0]  69 | 16.6 (1.8)  13.0  [0.6; 80.9]  78 | 31.0 (2.5)  26.8  [3.8; 243.0]  87 |
|  | total N (g kg^-1^) | Mean (SE)  Median  [min; max]  CV (%) | 2.3 (0.1)  2.1  [0.7; 7.9]  41 | 2.4 (0.1)  2.1  [0.6; 6.4]  55 | 0.8 (0.1)  0.7  [0.03; 6.6]  117 | 2.5 (0.2)  2.0  [0.3; 16.0]  79 |
|  | C:N | Mean (SE)  Median  [min; max]  CV (%) | 11.1 (0.2)  10.6  [8.2; 22.6]  19 | 12.1 (0.3)  10.3  [8.9; 24.4]  28 | 25.4 (1.3)  23.4  [9.0; 50.8]  37 | 12.9 (0.4)  11.6  [7.0; 29.5]  30 |
|  | CaCO_3_ (g kg^-1^) | Mean (SE)  Median  [min; max]  CV (%) | 1.5 (1.3)  0  [0.0;168.0]  990 | 36.0 (7.9)  0  [0.0; 460.0]  230 | 0.3 (0.2)  0  [0.0; 11.3]  470 | 187.9 (16.9)  153  [0.0; 739.0]  100 |
|  | K (g kg^-1^) | Mean (SE)  Median  [min; max]  CV (%) | 0.4 (0.02)  0.3  [0.1; 1.4]  66 | 0.4 (0.02)  0.3  [0.1; 1.1]  53 | 0.06 (0.01)  0.04  [0.0; 0.3]  107 | 0.5 (0.03)  0.4  [0.1; 1.8]  61 |
|  | Mg (g kg^-1^) | Mean (SE)  Median  [min; max]  CV (%) | 0.9 (0.1)  0.7  [0.1; 12.1]  141 | 0.8 (0.04)  0.7  [0.1; 2.9]  57 | 0.2 (0.1)  0.1  [0.02; 3.0]  177 | 1.4 (0.1)  0.9  [0.3; 10.4]  107 |
|  | Assimilable P (g kg^-1^) | Mean (SE)  Median  [min; max]  CV (%) | 0.1 (0.01)  0.09  [0.0; 0.3]  66 | 0.04 (0.003)  0.03  [0.0; 0.2]  97 | 0.02 (0.006)  0  [0.0; 0.2]  220 | 0.03 (0.004)  0.02  [0.0; 0.3]  140 |

**Table S1. Summary statistics of regions characteristics.** PNE: Particular Natural Ecosystems, SE: standard error of the mean

This table summarizes the summary statistics for each environmental variable in each region.
